# Supplementary material for: The Role of Cullin-RING Ligases in Striated Muscle Development, Function, and Disease
Source: Int J Mol Sci. 2020 Oct 26;21(21):7936. doi: 10.3390/ijms21217936 (PMC7672578; doi:10.3390/ijms21217936)

**Supplemental File 2** - unmodified immunoblot images for blots shown in Figure 7.

## Blots shown in Figure 7B

(antibodies and sample loading is marked on the blots; please note that the order of samples is reversed; +/- indicates the first sample: +/- cre-; arrow indicates bands shown in the figure )

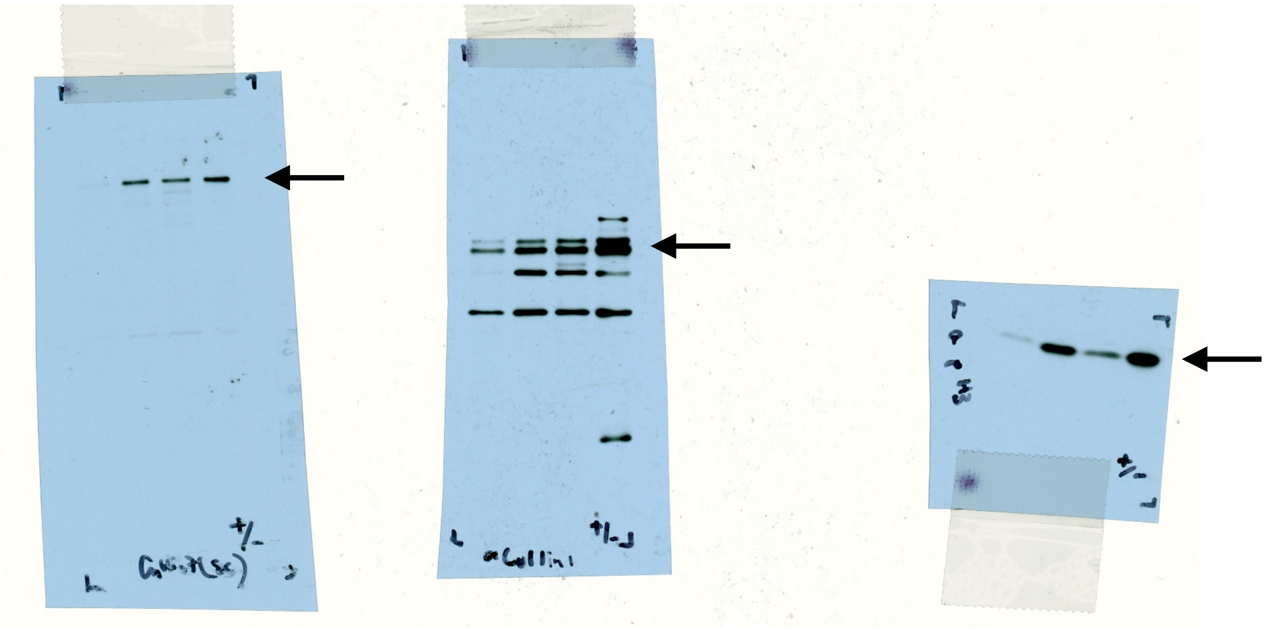

**Blots shown in Figure 7F**
(antibodies and sample loading is marked on the blots; arrow indicates bands shown in the figure and directionality of sample loading)

- Cullin 1

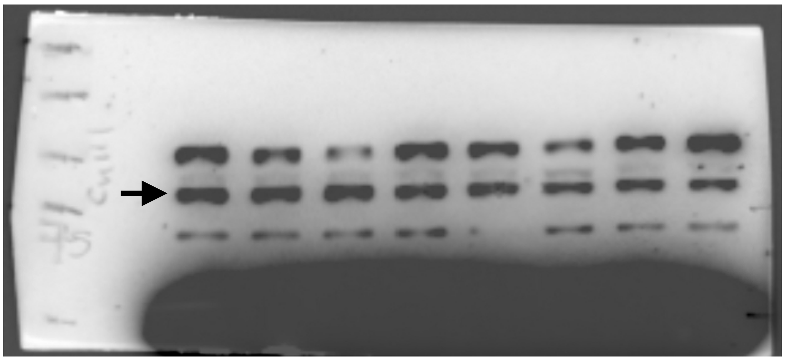

- Cullin 3

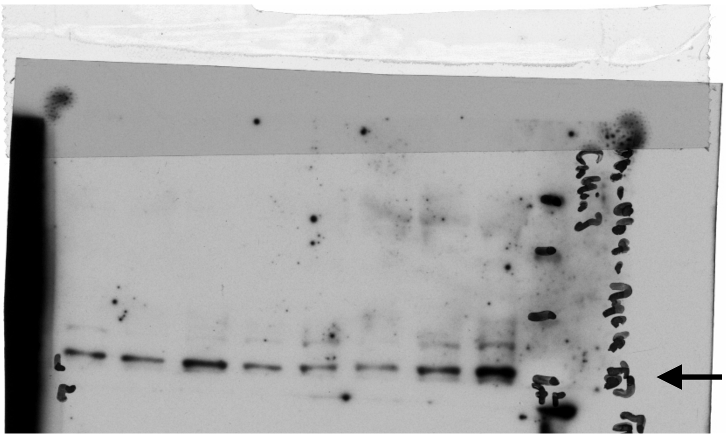

- Cullin 7
  please note, first lane is the molecular weight marker

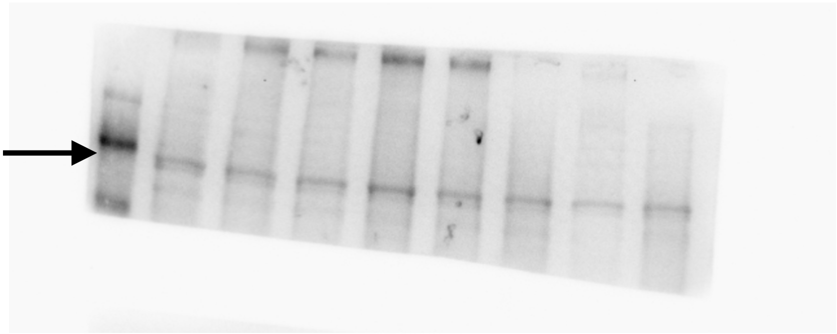

Supplement: Supplementary file 1 [file ijms-21-07936-s001.zip › Supplemental File 2.pdf]
